# From Stripes to a Beating Heart: Early Cardiac Development in Zebrafish

**DOI:** 10.3390/jcdd8020017

**Published:** 2021-02-10

**Authors:** Cassie L. Kemmler, Fréderike W. Riemslagh, Hannah R. Moran, Christian Mosimann

**Affiliations:** Department of Pediatrics, Section of Developmental Biology, University of Colorado School of Medicine and Children’s Hospital Colorado, Anschutz Medical Campus, Aurora, CO 80045, USA; cassie.kemmler@cuanschutz.edu (C.L.K.); frederike.riemslagh@cuanschutz.edu (F.W.R.); hannah.moran@cuanschutz.edu (H.R.M.)

**Keywords:** zebrafish, heart, development, cardiovascular, congenital heart disease, cell fate, lateral plate mesoderm

## Abstract

The heart is the first functional organ to form during vertebrate development. Congenital heart defects are the most common type of human birth defect, many originating as anomalies in early heart development. The zebrafish model provides an accessible vertebrate system to study early heart morphogenesis and to gain new insights into the mechanisms of congenital disease. Although composed of only two chambers compared with the four-chambered mammalian heart, the zebrafish heart integrates the core processes and cellular lineages central to cardiac development across vertebrates. The rapid, translucent development of zebrafish is amenable to in vivo imaging and genetic lineage tracing techniques, providing versatile tools to study heart field migration and myocardial progenitor addition and differentiation. Combining transgenic reporters with rapid genome engineering via CRISPR-Cas9 allows for functional testing of candidate genes associated with congenital heart defects and the discovery of molecular causes leading to observed phenotypes. Here, we summarize key insights gained through zebrafish studies into the early patterning of uncommitted lateral plate mesoderm into cardiac progenitors and their regulation. We review the central genetic mechanisms, available tools, and approaches for modeling congenital heart anomalies in the zebrafish as a representative vertebrate model.

## 1. Introduction: Zebrafish to Study Earliest Heart Development

Powerful genetic tools, rapid external embryonic development, optical transparency of the embryo, and a high number of offspring render the zebrafish (*Danio rerio*) an increasingly common vertebrate system for studying development and for modeling human disease [1,2]. Although seeming structurally simple with only two main chambers, the atrium and ventricle, the zebrafish heart is by no means a primitive heart, as it has adapted to teleost physiology over millions of years from our last common chordate ancestor [3,4]. Accordingly, early zebrafish heart development progresses via mechanisms shared among all chordate hearts, including the mammalian heart, rendering zebrafish a suitable model to study common principles of early heart formation [5,6]. As illustrated throughout our review, this conservation makes zebrafish accessible to investigate both the developmental mechanisms that form a vertebrate heart, as well as genetic causes and consequences of cardiovascular anomalies.

Beyond the unique opportunity to observe heart development in optically transparent embryos, zebrafish mutants with cardiac defects have informed our understanding of the molecular pathways and cell fate control underlying early heart formation [6]. Derived from large forward-genetic screens and serendipitous observations alike, now-classic cardiac mutants include *cloche* (*clo*) that lack endothelial and hematopoietic lineages and develop larger, progressively failing hearts [7]; *casanova* (*cas*), *bonnie and clyde* (*bon*), and *two of heart* (*toh*) that all result in the formation of two beating hearts or cardia bifida [7,8,9,10]; *weiches herz* (*whz*) with diminished cardiomyocyte proliferation [11]; or *heartstrings* (*hst*) and *hands off* (*han*) that both develop severely perturbed hearts and abnormal pectoral fins (homologous appendages to our forelimbs) [12,13]. Identification of the genes affected in these and other mutants has provided unprecedented insights into seemingly zebrafish-specific as well as deeply conserved mechanisms of early heart development. Combined with increasing information about conserved gene expression patterns in early cardiac development, the observation of developmental stages in both wildtype and diverse mutant backgrounds in zebrafish has complemented parallel work in other vertebrate model systems.

**Figure 1:**
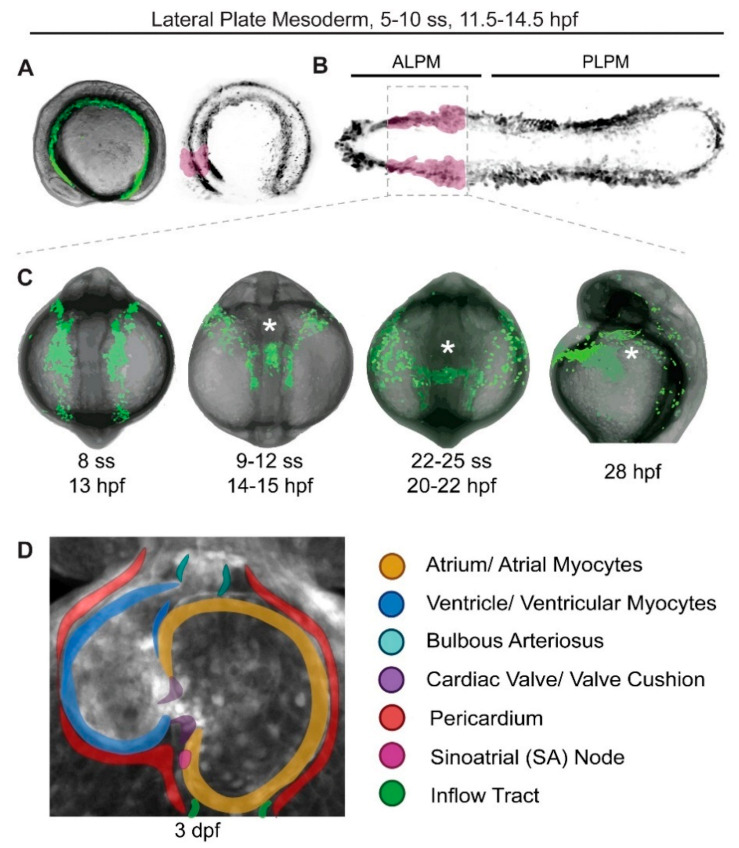
Earliest stages of cardiac development in zebrafish. Stages of zebrafish heart formation from lateral plate mesoderm (LPM). (**A**,**B**) LPM during early somitogenesis on three-dimensional (**A**) and virtually flat-mounted (**B**) zebrafish embryo, anterior to the left. The prospective bilateral heart field is marked in magenta within the anterior LPM (ALPM). (**C**) Stages of heart field convergence at the midline, formation of the cardiac disc and cone, migration to the left, and early heart tube stages; maximal projection of z-stack of *drl:EGFP* transgenics [24], anterior (head) to the top, with the embryo curved over the yolk at this stage (tail end behind the yolk in the first three panels, out of view in the right-most panel). Note how the bilateral ALPM migrates to the midline and forms the leftward-moving heart tube (asterisks). (**D**) 72 hpf (3 dpf) zebrafish heart with all major parts color-coded, anterior/rostral to the top; greyscale max projection of z-stack of *ubi:Zebrabow* transgenics overlaid with annotation of individual structures [25]. See text for details, images not to scale.

As in all vertebrates, zebrafish cardiac progenitors emerge in the anterior bilateral stripes of the lateral plate mesoderm (LPM) in close coordination with their surrounding cells [14,15,16,17,18,19] (Figure 1A). The initially bilateral cardiac progenitors migrate medially from either side to form the intermediate cardiac crescent or disc that then shapes the primitive heart tube, a process that is highly accessible to imaging and perturbations in zebrafish [5,20] (Figure 1B,C). As the body’s first asymmetric organ, the extruding heart tube migrates towards the left in a process referred to as leftward jog around 21–23 h post-fertilization (hpf) [21,22,23]. Consisting of the atrium and ventricle, the linear heart tube begins synchronized contractions around 24 hpf and supports a rudimentary circulatory system with red blood cells (erythrocytes) flowing through the dorsal aorta and cardinal vein, the latter of which only fully connects to the heart later (Figure 1).

By 3–5 days post-fertilization (dpf), the zebrafish heart principally features an inflow tract (IFT) portion, atrium, a trabeculated ventricle separated by a tricuspid valve [20,26], an outflow tract region encompassing the distal ventricle and ending in the smooth muscle-based bulbus arteriosus (BA) that acts as pressure capacitator [27,28], and a complex functional conduction system regulated by the cardiac pacemaker of the sinus venosus at the IFT [29] (Figure 1D). This astonishingly rapid and accessible development in a vertebrate embryo has enabled previously unprecedented experiments to understand the earliest processes leading to cardiac progenitor patterning within the LPM and initial morphogenesis of the heart.

## 2. Earliest Developmental Steps towards Heart Formation

Across vertebrates, heart progenitors arise among the other cardiovascular progenitors within the emerging LPM, a dedicated mesodermal progenitor domain that forms at the lateral edge of the developing embryo [19,30,31]. In zebrafish, the prospective LPM is detectable from gastrulation stages by expression of *draculin* (*drl*), which is induced by the broader acting, early mesendoderm regulators Eomes, FoxH1, and Mixl1 [32] (Figure 1). The positioning of prospective cardiac progenitors contributing to the atrium and ventricle within the earliest LPM has been mapped in great detail by cell labeling using caged fluorophores during gastrulation stages [33]. After gastrulation, the myocardial and endocardial progenitors migrate as part of the laterally converging anterior LPM (ALPM), possibly restricted by signals from adjacent LPM progenitors such as anterior vascular precursors and posterior limb precursors [34,35]. Contrary to human, mouse, and *Ciona,* where Eomes activity triggers the expression of Mesp1 in the prospective cardiac progenitors within the ALPM [36,37,38,39], expression of Mesp1 orthologs seems irrelevant for the formation of zebrafish cardiac progenitors, which follow a currently unknown mode of induction [40,41]. Nonetheless, downstream of Mesp1, mammalian heart progenitors turn on expression of the key cardiac transcription factor Nkx2.5 [42,43,44], and expression of *nkx2.5* within the zebrafish ALPM includes the cardiac progenitors as the earliest mark of their emergence to date [45]. As *Drosophila* uses the *nkx2.5* ortholog *tinman* to control heart development, Nkx2.5 activity is an ancient feature of early heart formation [46,47].

During somitogenesis, the ALPM patterns into overlapping progenitor fields expressing conserved cardiac, endothelial, and hematopoietic genes including *gata4/5/6*, *hand2*, *tbx1*, *tbx5*, *mef2ca/b*, *kdrl*, *cdh5*, *scl*, *lmo2*, and *pu.1* [19,48,49,50,51,52,53,54,55,56]. Subsequently, *kdrl*- and *cdh5*-expressing endocardium precursors migrate towards the midline where they fuse to form the inner endothelial lining of the heart, coordinated with cardiomyocyte precursors that form the heart muscle [15,56]. The close proximity of cardiac progenitors to ALPM progenitors that give rise to cranial endothelium and myeloid cells in zebrafish further hints at the possibility of multi-potent progenitor cells, akin to posterior LPM-residing hemangioblasts that act as joint endothelial and erythrocyte progenitors [57,58].

At approximately the 14 somite stage (ss, 16 hpf), first cardiomyocyte differentiation becomes detectable by expression of *myosin light chain polypeptide 7* (*myl7*, previously called *cmlc2*), a commonly used marker of differentiating myocardium [59,60]. Additionally, by approximately 19 hpf, progenitor cells begin expressing *ventricle myosin heavy chain* (*vmhc*) and a section of more laterally positioned cells express *atrial myosin heavy chain* (*amhc* or *myh6*), prefacing the regionalization of the heart field into precursors of the future chambers while the ALPM converges at the midline [60,61]. Fusion of the migrating bilateral heart field cells at the midline forms the cardiac disc with endocardial progenitors located in the middle, flanked by ventricular and atrial progenitors at the periphery [15,56,60,62]. This convergence at the embryo midline is dependent on proper endoderm integrity: disruption of endoderm formation, as impressively displayed in the *sox32* mutant *casanova* (*cas*) that is entirely devoid of endoderm, results in failed midline migration of ALPM and the formation of two hearts at the initial position of the bilateral cardiac progenitors [8,9,63,64]. Curiously, *cas,* as well as other endoderm-affecting mutants including *mixl1* (*bon*) [7,65], still form beating hearts, suggesting that intrinsic cues within the ALPM progenitors are sufficient for basic heart patterning. Nonetheless, how the endoderm supports the merging ALPM progenitors to form the heart remains to be fully revealed. One hint comes from PDGF signaling that has specifically been linked to ALPM midline migration, with endoderm generating PDGF ligand and myocardial precursors expressing its receptor [66].

The cardiac disc elongates to form a cone and subsequently the linear heart tube (Figure 1C) [6]. By 48 hpf, the zebrafish heart has looped and consists of the atrium and ventricle separated by the atrio-ventricular canal (AVC) and lined by the endocardium [59]. Beyond an increasing anterior-posterior regionalization of gene expression programs within the heart tube [67], the heart already features intrinsic asymmetry beyond its left-sided migration, as detectable by dorsal restriction of *meis2b* expression predominantly in the atrium [68]. Subsequent maturation processes over the first 3–5 dpf include the formation of the epicardium, development of valves at the AVC, OFT, and IFT, progressive trabeculation of the ventricular myocardium, and full differentiation of the BA into a pressure capacitator to attenuate the outflow of blood into the gills (Figure 1D) [26,28,69,70,71,72,73,74,75,76]. This striking pace of heart development in zebrafish provides a versatile platform to study the basic principles and structures involved in forming a functional vertebrate heart.

## 3. Labeling and Live Imaging of Heart Progenitors

Within the LPM, the emergence, patterning, and migration of cardiac progenitors is trackable in real time through fluorescent transgenic reporters based on isolated gene-regulatory elements. Transgenic zebrafish have become widely applied in vivo tools thanks to easily available and universal plasmid collections, genetic methods to track and perturb cell lineages, and advances in live imaging of developing embryos using confocal, spinning disk, or light sheet microscopy [77,78,79,80,81,82]. Previously confined to cell-based labeling, transgenic marking of the cardiovascular lineage-forming LPM and its divergent cell lineages has enabled live imaging and isolation of the earliest stages of cardiac progenitor formation. The regulatory elements of the zebrafish gene *drl* [55] established an early genetic marker for the developing LPM starting from 8 hpf onwards and that subsequently labels cardiovascular lineages including the prospective cardiac progenitors (Figure 1A,B) [24]. Cre/*lox*-based lineage tracing using *drl*-based transgenic reporters and live imaging of the emerging LPM from gastrulation stages have provided new insights into the context of heart development embedded within its adjacent LPM lineages [32,83,84]. Gene-regulatory elements of the mouse *Smarcd3* gene [85] have been successfully harnessed to generate an early LPM-marking transgenic reporter in zebrafish that includes cardiac progenitors [86]. Fluorescent color variants of transgenic reporters based on subsequently expressed cardiac genes have generated a repository of tools for the field, including reporters based on *myl7*, *kdrl*, *elnb*, *tbx1*, *nppa*, and more that have unveiled distinct processes of heart development [87,88,89,90,91,92,93]. Larger BAC-based transgenes have especially provided groundbreaking reporters for key cardiac genes including *nkx2.5*, *gata5*, *hand2*, and *tbx5* that were applied in the study of early ALPM partitioning and patterning of the heart [83,88,94,95]. With increased efforts to discover gene-regulatory elements and the prospect of targeted knock-in approaches, we are in a highly productive time for the generation of new transgenic zebrafish reporters to visualize heart development and related processes, as well as to unravel the upstream regulation of unique gene-regulatory programs driving heart formation.

## 4. Heart Formation from the First and Second Heart Fields

**Figure 2:**
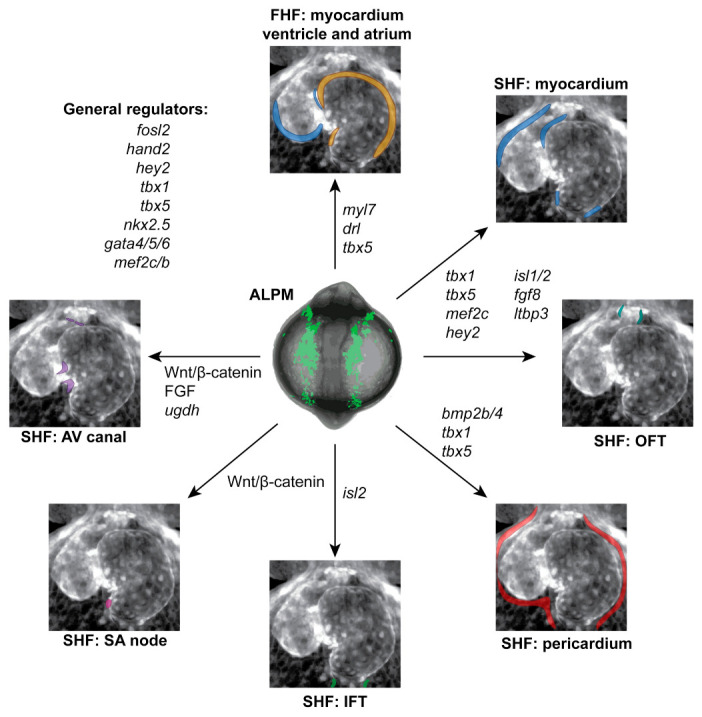
Genes contributing to early heart development in zebrafish. Overview of select genes and pathways involved in the formation and patterning of individual structures of the zebrafish heart, as representative at 3 dpf. Individual parts of the heart are color-coded corresponding to Figure 1. See text for details.

As outlined above, the early heart tube forms from bilateral ALPM following the cardiac disc, cone, and then the linear heart tube stages. Principally, the early heart consists of an endocardial lining surrounded by myocardium that is shaped into the atrium and ventricle, with a dedicated venous pole or IFT and an arterial pole or OFT capped off by the BA composed of smooth muscle (Figure 2). A large body of evidence has documented that vertebrate cardiomyocytes emerge from two distinguishable types of ALPM progenitors deemed the first and second heart field (FHF and SHF, respectively). Originally described in mammals, the FHF chiefly forms the left ventricle myocardium and connects the heart to systemic circulation, while the SHF forms the atria and right ventricle for lung circulation [96] (Figure 2). Analogous in zebrafish, FHF progenitors originate in the ALPM and migrate to the midline by the 18 somite stage (18 hpf) to form the primary, linear heart tube (Figure 1). Given the deep conservation of FHF and SHF lineages across chordates, including in *Ciona*, the discovery of SHF-associated progenitors in zebrafish has expanded the context for their adaption and contribution to the heart [3,97,98,99].

Transgenic tools have proven instrumental in uncovering the conserved SHF in zebrafish. Differential maturation of the fast-folding EGFP and slower dsRED expressed under transgenic control of the *myl7* promoter revealed that cardiomyocytes at both the arterial and venous poles are later-differentiating populations [92]. Photoconvertible fluorescent *myl7:KikGR* revealed more precisely the timing of SHF-liked myocardium addition at the arterial pole between 24 and 34 hpf and the contribution of Mef2c to this process [93]. Caged fluorophores have been deployed as cell tracing tools to identify SHF precursors located posterior to the arterial pole of the early heart tube that contribute to the distal myocardium of the ventricle and the smooth muscle of the BA [33,100]. Select transgenic reporters also discriminate FHF from SHF. BAC-based reporters using the *ltbp3* locus were found to be selectively active in the late-differentiating ventricular and OFT portion of the heart [88], while late somite-stage expression of transgene reporters based on *drl* and *tbx5a* is confined to FHF lineages [24,83]. FHF labeling by *drl*-based reporters also revealed that SHF addition to both poles of the primitive heart tube is independent of endocardium formation, as shown in *clo* mutants that lack the endothelium and blood lineage [24]. Light sheet-based live imaging using *tbx1*-based transgenic reporters captured the continuous addition of progenitors to the forming ventricle and OFT region as a stream of FHF and SHF cells from the bilateral LPM during heart tube elongation [89]. These collective observations are complemented by recent live imaging in developing chicken and mouse embryos [101,102,103], further underlining that zebrafish use deeply conserved mechanisms to coordinate early heart development that even our tunicate relatives deploy [104].

## 5. Developmental Signaling in Early Heart Development

The FGF, BMP, TGF-beta, retinoic acid (RA), and Wnt pathways are major developmental signaling cascades that have all been implicated in diverse aspects of early vertebrate heart formation. Mutants affecting FGF signaling have been among the earliest isolated heart anomalies in zebrafish, as illustrated with the mutant for *fgf8* (*ace*) that features predominant ventricle defects [54]. FGF signaling is deployed at various stages during mesoderm and cardiovascular fate patterning, starting with dorso-ventral patterning during gastrulation, to drive cardiac fate and restrict anterior endothelial-hematopoietic fates in early somitogenesis, and in controlling *nkx2.5* and *gata4* expression in cardiac precursors [54,105,106,107]. Perturbations of FGF signaling during the medial migration of the ALPM progenitors reduce ventricle size and inhibit BA formation, possibly suggesting a spatial or temporal sensitivity to FGF signaling in the anterior-posterior patterning of the OFT region [89,107]. Pulsed chemical FGF signaling perturbation after 20 hpf leads to reduced cardiomyocyte addition to the arterial pole of the heart, and inhibition of FGF signaling after heart cone formation results in loss of *mef2cb* and *vmhc* expression in the developing ventricle [92,93]. At 3–5 dpf, FGF signaling continues to contribute to maintaining the integrity of the myocardial wall downstream of Klf2 and to maintaining ventricular identity [108,109]. Further, *hey2* has been charted as a downstream FGF target that controls the proliferative capacity of cardiac progenitor numbers, with extended growth of the heart in *hey2* mutants [110]. While these results implicate FGF signaling in controlling SHF, the extent of the cell-autonomy of this process remains to be determined. Additionally, *fgf8a* expression is perturbed in embryos with an excess of RA signaling caused by loss of the RA-degrading enzyme Cyp26, leading to a failure in SHF progenitor addition to the OFT that is partially rescued when FGF signaling is restored [111]. The excess of RA causes a shift in contribution to the pharyngeal arch arteries instead of the OFT and a loss of progenitor to the heart tube [111]. Interconnecting two key signaling pathways, these data add to the model that RA acts to attenuate FGF responses during heart and adjacent fin field formation [112,113].

Beyond their key involvement in dorso-ventral patterning and LPM initiation, the BMP/TGF-beta/Nodal family of signaling molecules has been repeatedly linked to various aspects of early heart formation across vertebrates [114]. Active Bmp signaling drives general differentiation of cardiomyocytes, while chemical perturbation of Bmp signaling at 24 to 48 hpf leads to an increase in BA size at the expense of arterial pole myocardium, suggesting that Bmp patterns the zebrafish SHF by defining myocardial versus smooth muscle fates [92,100]. Asymmetric Nodal signaling on the left and Bmp signaling act in parallel to establish zebrafish cardiac laterality and correct heart looping [23,115,116]. From 48 hpf onwards, endocardial Notch signaling has been implicated in triggering *bmp2b* and *bmp4* expression that prompts pericardium progenitor cluster formation, and zebrafish lacking the Bmp receptor Acvr1l fail to form a proepicardium [70,117]. The forkhead transcription factor FoxH1 mediates the cell responsiveness to Bmp and cooperates with Smads activated downstream of Nodal signaling to control its target genes [116,118,119,120]. More broadly, conserved FoxH1 targets include genes encoding signaling ligands such as *fgf8, fgf3,* and *wnt11* [118,119]. Both the expression and activity of the secreted TGF-beta inhibitor Ltbp3 have been linked to modulating the patterning of the OFT: *ltbp3* morphants show reduced TGF-β signaling activity and SHF proliferation defects [88]. Moreover, *lftbp3* has been shown to be a downstream target of *nkx2.5* in zebrafish, adding *ltbp3*-mediated modulation of TGF-β among the downstream effectors of *nkx2.5* activity [121]. Together, the data on the influences of BMP/TGF-beta/Nodal signaling and their downstream Smad transcription factors underline various spatio-temporal contributions to early heart development. How this key family of signaling processes intersects and cooperates with individual cardiac transcription factors remains a widely investigated topic in the field.

Wnt signaling encompasses at least three distinct and widely deployed developmental signaling cascades that influence several aspects of early heart formation, dependent on the developmental timing of its deployment and the involved ligands and receptors [122,123,124,125,126]. Canonical Wnt signaling, also referred to as Wnt/β-catenin signaling due to its key downstream transcriptional effector β-catenin, promotes LPM by supporting ventral and lateral fates during gastrulation. Excess Wnt signaling during later gastrulation and early somitogenesis promotes the expansion of FHF progenitors and reduces SHF progenitors within the ALPM [122,127]. Canonical Wnt signaling is involved in AV canal formation and deployed to induce the AV valve by acting upstream of FGF signaling [128,129]. Non-canonical Wnt signaling controls planar cell polarity effects on tissues, a process involved in numerous embryonic processes including axis elongation, epithelia organization, and organogenesis. Zebrafish mutants for the non-canonical Wnt ligands Wnt11, Wnt5a, and Wnt4 show cardia bifida and subsequent defects in heart tube formation due to issues with midline migration [130]. Later on, Wnt11 and Wnt5 ligands have especially been linked to the initial coordination of cardiac conductivity by patterning the electric gradient in the myocardial syncytium [131].

The Hedgehog pathway, triggered in zebrafish by the orthologs Sonic Hedgehog (encoded by *shh*), Indian Hedgehog (*ihh*), Desert Hedgehog (*dhh*), and Tiggy-winkle Hedgehog (*twhh*), contributes to a variety of developmental processes predominantly after gastrulation [132]. Downstream of Wnt, *shh* expression in the midline is essential for heart asymmetry [133]. Genetic and pharmacological perturbations of Hedgehog signaling have been linked to several effects on the developing heart, including reduced cardiomyocyte numbers, severely interrupted OFT formation, and diminished endocardial morphogenesis in the mutant *slow muscles omitted* (*smu*) that perturbs the Hedgehog pathway effector Smoothened [100,134,135] and upon conditional loss of Shh using a unique Cre/*lox*-controlled allele [136]. As with other signaling pathways, the origin of the Hedgehog signal(s) during individual stages of early heart development remains incompletely charted. Curiously, heart formation is sensitive to Shh that is possibly coming from surrounding organs, with loss of key endocardial markers already in early to mid-somitogenesis upon pathway inhibition [135]. Similarly, *Gata4* control during heart development in mice has been implicated as downstream target of notochordal Shh secretion [137], providing a curious anatomical connection between disparate organs during their embryonic morphogenesis. Taken together, these results underline that vertebrate heart development depends on a multitude of signaling pathways of which timing and tissue-specific induction are crucial to provide correct formation of the heart.

## 6. Connecting Individual Players to Developmental Interactions

Genetic analysis has revealed genes involved in individual aspects of early heart morphogenesis, in particular ventricle and OFT formation. The T-box factors Tbx1, Tbx5, and Tbx20 contribute to early heart development across vertebrates [138]. Tbx1 has been repeatedly implicated in vertebrate SHF differentiation, and impaired *tbx1* function perturbs heart development including SHF progenitor proliferation in zebrafish [3,139,140,141]. The upstream regulation of *tbx1*, as of many other cardiac transcription factors, remains incompletely understood [142]; one piece to the puzzle is the finding that impaired *hdac1* or *cyp26* function, leading to increased retinoic acid receptor (RAR) activity, triggers increased expression of Ripply3, which in turn acts as a repressor of T-box factor genes including *tbx1* [143]. Zebrafish *tbx5* mutants prominently recapitulate the joint cardiac and forelimb defects found in human *TBX5*-associated Holt–Oram syndrome patients, the original paradigm of so-called heart-hand anomalies [12,144,145,146,147]. Beyond its well-documented role in early ALPM patterning towards cardiac and forelimb progenitors, loss of *tbx5* causes an expansion of FHF contribution to the heart, while *pitx2* perturbation reciprocally results in reduced FHF [24].

The broadly acting cardiac Gata factors Gata4/5/6 have also been repeatedly implicated as key regulators of SHF-involving processes; their exact individual contributions remain to be further elucidated, a challenge due to functional redundancy and crosstalk [148,149,150,151,152]. In mice, the cardiac Gata factors have been implicated at various levels of heart patterning: as downstream and feedback effectors of Shh signaling, downstream of *Nkx2.5*, upstream of *Mef2c*, and in cross-regulating each other such as Gata4 controlling Gata6 activity during SHF proliferation, septation, and outflow tract formation [137,153,154,155]. In zebrafish, *gata4/5/6* already contribute to early mesendoderm patterning, complicating interpretations of later phenotypes [54,150,156]. Analysis of combinatorial *gata4/5/6* morphants and mutants suggests that cardiac specification depends primarily on an overall dosage of these cardiac Gata transcription factors rather than a specific gene [148,150,151,152,157,158]. *gata6* seems to primarily regulate the differentiation of ventricular cardiomyocytes and ventricle morphogenesis through regulation of both the FHF and SHF [151].

The homeobox-containing transcription factor Nkx2.5 is universally required in vertebrates at initial stages of heart progenitor determination in the ALPM, for subsequent cardiomyocyte differentiation for chamber formation and identity, and later in the SHF to complete initial heart morphogenesis [121,159]. Nkx2.5 acts synergistically and at least in parts redundantly with its paralog Nkx2.7 in controlling arterial and venous pole SHF contribution, adding another level of fine-tuning [109,160]. Single cell RNA-sequencing of *nkx2.5*-positive cells charted multiple subpopulations of SHF progenitors and differentiating ventricular cardiomyocytes at the arterial pole in zebrafish embryos [161], underscoring the broad involvement of *nkx2.5* expression during multiple stages in cardiac development. More work is warranted to unlock the secrets of Nkx2.5’s ancient history of contributing to heart formation [47,162,163].

Of particular interest for zebrafish SHF development are the Islet family transcription factors Isl1 and Isl2, the study of which has revealed new layers in SHF differentiation and proliferation. In mice, *Isl1* gene expression demarcates SHF progenitors and its loss results in atrial and ventricular defects of SHF-derived lineages [164,165,166,167]. Consequently, several studies have addressed the expression and functional involvement of the *isl* genes in zebrafish SHF formation, with *isl1* becoming a frequent marker for the highly migratory SHF-derived progenitors at the venous pole and inflow tract structures [92,100,168]. As is common for gene paralogs in zebrafish as the result of the teleost-specific whole-genome duplication in the last common ancestor of bony fishes [169], the Isl paralogs *isl1a*, *isl1b*, *isl2a*, and *isl2b* have acquired regionalized functions, and the OFT and arterial pole require *isl2b* for their proper formation [170]. These findings further revealed *isl2b* as acting upstream of various cardiac transcription factor genes including *mef2c* genes, *hand2*, and *tbx20*, connecting the activity of regional progenitor programs to broad-acting effectors [170]. The LIM domain-binding protein ldb1 regulates differentiation of the *isl1*-expressing cardiomyocytes at the venous pole [171], while the LIM domain protein Ajuba restricts Isl protein activity as a downstream effector of RA signaling [168] and Nkx2.5 and Nkx2.7 have been implicated in repressing *isl1* at the venous pole [172]. These studies reveal a fine-grained early control of ALPM progenitors that form the heart that goes beyond a merely FHF versus SHF patterning choice. Together, this striking regionalized gene regulation suggests that molecular differences between FHF and SHF progenitors, despite their seemingly fluid transition in forming the heart, are engrained early on in development by yet-to-be revealed mechanisms and evolutionary origins.

## 7. The Cardiopharyngeal Field as Context for Heart Formation

While traditionally studied as an individual organ, the heart develops in close coordination with other LPM-derived cell fates, most apparent with endothelial and hematopoietic lineages. Detailed lineage mapping over the past two decades has painted a more integrative picture of heart development and its connection with other organ systems, some of which are not immediately apparent from the adult body plan. Pioneering work in mice has revealed that the SHF shares lineage origins with select muscles in the neck and head [173]. Extensive research across chordate models has unveiled that cardiac and branchiomeric muscle progenitors share a joint developmental origin, embedding the heart field within a broader progenitor pool deemed the cardiopharyngeal field (CPF) [174,175]. The CPF includes FHF and SHF progenitors, as well as branchiomeric progenitors residing in the pharyngeal mesoderm [166,176,177]. Supporting an ancient evolutionary lineage relationship is the expression of homologs of *Nkx2-5* and *Isl1* in overlapping territories in anterior and ventral mesoderm domains reminiscent of the anterior LPM in more basal vertebrates such as lampreys, gnathostomes, cyclostomes, and amniotes, and the development of CPF lineages including FHF, SHF, and syphon muscles in the chordate *Ciona* [97,178,179,180,181,182,183,184]. Nonetheless, the descriptions of anterior or head-associated mesodermal lineages remain incompletely established, and whether the CPF is entirely embedded within the ALPM remains unclear. Further, which endothelial lineages in the head and pharyngeal arches should be considered part of the CPF awaits further definition.

The accessibility of zebrafish development to imaging and the wealth of both available mutants and validated knockdown reagents further contributed to our current grasp of CPF-associated formation and patterning. The overall gene expression pattern of ALPM-associated lineages is well-documented for various timepoints, as well as territories in the ALPM with cardiac, endothelial, and hematopoietic potential [34,89,183,185]. *tbx1* mutants develop widely documented defects consistent with perturbed CPF lineages, including SHF and craniofacial anomalies [140,186]. Loss of endothelial and hematopoietic lineage specification in the ALPM in *clo* mutants results in ectopic expression of myocardium-associated genes rostrally of their normal territory, possibly involving *etv2*/*etsrp* and *scl*/*tal1* as also found in mammals [34,56,187]. Loss of *tbx1* results in failed specification of progenitors involved in OFT, head muscle, and pharyngeal artery formation that co-express *tbx1*, *nkx2.5*, and the TGF-beta/Activin ligand gene *gdf3* [185,188]. Canonical Wnt signaling has been linked to cell-autonomous inhibition of pharyngeal muscle fates at the expense of general cardiac progenitor fates in zebrafish [127]. Similarly, as described above, FGF signaling influences the timing and ultimate quantity of cells contributing to SHF-derived lineages [89,92,107], which echoes the influence of FGF signaling on CPF lineage differentiation in *Ciona* [189,190]. Recent single-cell sequencing and mutant analysis implicates *gata4/5/6* in the segregation of cardiac versus pharyngeal fates, with perturbed Gata5/6 function resulting in reduced cardiac and increased pharyngeal progenitors, which mirrors the situation described for *Ciona*, underlining another deeply conserved regulatory mechanism of CPF patterning [157]. Overlaying the CPF concept with current models of ALPM patterning and myocardial and endocardial lineage formation provides a powerful context to understand the evolution, development, and disease context of early heart formation.

## 8. Modeling Early Causes of Congenital Heart Disease in Zebrafish

**Figure 3:**
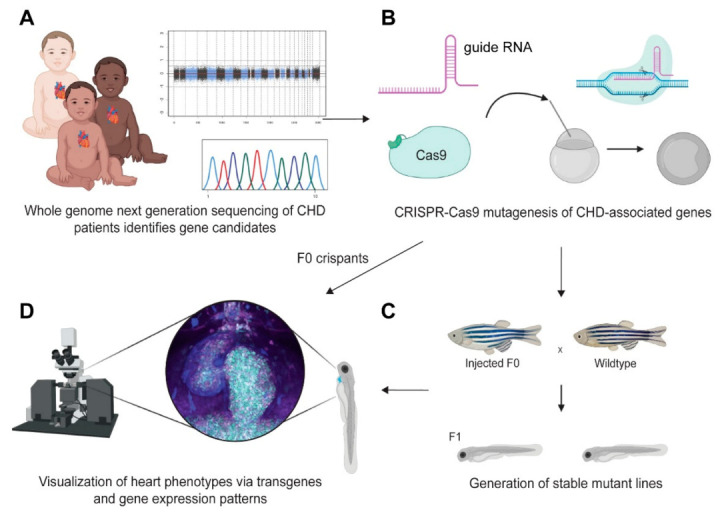
Zebrafish as discovery platform for congenital heart disease genes. General workflow for linking patient mutations to cardiac phenotypes using zebrafish. (**A**) Whole-genome sequencing or other approaches inform about possible candidate genes responsible for congenital heart anomalies. (**B**) CRISPR-Cas9-based mutagenesis of the candidate genes by microinjection enables the generation of germline mutations towards isolating mutant alleles, as well as first phenotype assessment and screening in F0 embryos (crispants). (**C**) Establishment of germline mutants for subsequent genetic follow-up of phenotypic consequences of candidate mutations. (**D**) Phenotype assessment of crispants or germline mutants via microscopy, assisted by transgenic labeling or staining for individual gene expression patterns (i.e., mRNA in situ hybridization, immunohistochemistry, and others). The fluorescent panel is a max projection of a triple transgenic zebrafish at 3 dpf carrying *drl:EGFP, myl7:CFP*, and *lmo2:dsRed* [24,88,198,199]. See text for details. Schematics generated with BioRender.

While zebrafish develop a beating heart within 24 hpf, zebrafish development can move on even without a functional circulatory system, as oxygen can reach embryonic tissues via passive diffusion [6,7]. In addition to chemical compound screening to find modulators of heart development with possible therapeutic use, this feature enables the study of cardiovascular development in embryos with mutations that cause early lethality in mammalian model systems. The rapid expansion of knockdown reagents based on morpholinos and mutants generated with a growing collection of tools has provided unique information about genes associated with heart development and human CHDs. The high efficiency of CRISPR-Cas9 mutagenesis in the zebrafish model system facilitates the genotype–phenotype association of CHD-associated loci and the generation of new disease models in zebrafish faster than ever before [191,192,193,194,195] (Supplementary materials, Table S1). The growing accessibility of next-generation sequencing in patients with CHDs holds promise to increase the detection of genomic alterations occurring in CHD patients [196,197]. While lacking anatomical features found in mammals including septation, a right ventricle and atrium, and the entire pulmonary circuit, in vivo testing of human CHD-associated mutations in zebrafish with its arsenal of readouts for basic heart and overall development establishes a first discovery platform for CHD mechanisms and co-morbidities affecting developmental processes beyond the heart (Figure 3, Supplementary materials, Table S1).

Several zebrafish mutants have provided the first functional link implicating a particular gene with a human disorder. Examples include the gene *titin* (*ttn.2*), which has been causally linked to familial dilated cardiomyopathy in the zebrafish *pickwick (pik)* mutant [200,201,202]; the zebrafish *jekyll (jek)* mutant with a loss-of-function mutation in the *ugdh* gene, which causally linked UDP-glucose 6-dehydrogenase to defective AV valve formation [203,204]; and the zebrafish mutant *gridlock (grl),* featuring a loss-of-function in the gene *hey2,* which has been causally linked to defects analogous to human aortic coarctation [205].

As well as the heart, the bilateral LPM also gives rise to blood, the kidneys, smooth muscles, limb connective tissue, and mesothelia [19]. These shared origins, including the CPF, have increasingly placed heart development in context with other early patterning and cell fate choices within the embryo. Zebrafish *hst* mutants affecting the T-box transcription factor Tbx5a recapitulate the principal phenotypes occurring in human Holt–Oram syndrome, which features anomalies in the development of the heart and the arms occurring in patients [145,206,207,208]. The zebrafish *van gogh (vgo)* mutant affects the transcription factor Tbx1, the loss of which presents both heart and craniofacial defects associated with human DiGeorge syndrome as hypothesized due to a perturbed Tbx1–Wnt regulatory axis in the CPF [139,140,186,209,210,211]. Implicated in left ventricular hypoplasia through genome sequencing of affected patients and association with repeatedly occurring *1q21.1* copy number variations (CNVs) [212,213,214], both zebrafish and mouse mutants for the nuclear Wnt signaling component Bcl9 display heart-looping defects and cardiac edema as well as craniofacial defects associated with disrupted Wnt/beta-catenin [215]. Beyond loss-of-function allele testing, knock-in and base-editing promise more precise recapitulation of human CHD-associated mutations, as demonstrated by recreating gain-of-function alleles of subunits constituting the ATP-sensitive potassium channel implicated in Cantú Syndrome [216]. These examples of zebrafish mutants revealing cardiac defects throughout development, within the morphology available in the model, underline the utility of using zebrafish as to associate human gene mutations with developmental heart defects and co-morbidities resulting from the integrated development of the heart within the CPF and broader LPM.

## 9. Conclusions and Future Challenges

While tetrapod-specific adaptations such as septation or formation of a pulmonary circuit are out of direct reach of study in the model, zebrafish have contributed to establishing basic concepts of early heart field emergence and patterning in vertebrates. Nonetheless, several aspects of cardiac fate initiation, heart field patterning, and initial morphogenesis await further research that can benefit from the zebrafish model’s capabilities. While the fundamental principles of early heart formation seem deeply conserved and shared across vertebrates, the discrepancy of how mammals and *Ciona* induce cardiac progenitors via Mesp1, while zebrafish do not [38,39,40,41], remains puzzling. Necessary but not sufficient, however, Mesp1 cannot be considered a master regulator for heart formation, and insights into how zebrafish induce cardiac progenitors within the ALPM could potentially unlock the general principles underlying the induction of heart formation from LPM progenitors.

Despite the research attention the SHF has enjoyed in recent years, several aspects of its biology remain unresolved. While various gene expression patterns and broader reporters have been used to map SHF progenitors within the ALPM, their specific location and dynamic migration remain to be fully determined. Single cell-based transcriptomics promises to reveal new details of SHF and earliest general heart field progenitors, as already approached in first studies [84,157,161]. Further, while broadly deemed SHF, to what extent its arterial versus venous pole-forming derivatives share a common progenitor pool in the early LPM is unclear, despite their regulatory differences [170,172]. At the molecular level, how the different SHF regulators interact transcriptionally with each other remains to be charted. Beyond Islet factors and Nkx2.5/7, examples include how the AP-1 factor Fosl2 connects to other SHF-regulating activities to promote the addition of these cells to the heart tube [217], or how *jnk1a* splice forms influence the ventricular myocardium upstream of *hand2* [218]. Notably, IFT formation from SHF progenitors has received considerably less attention than the development of the distal ventricle and OFT portion of the zebrafish heart, in part due to its at times disparate definitions and nomenclature [67,72,98,219]. Importantly, the IFT population of cells also gives rise to the cardiac pacemaker or the sinoatrial node (SAN), which regulates heartbeat frequency through recurring depolarization that drives contraction of the myocardium, and anomalies of the SAN can lead to sinus arrythmias and sudden death [29,67,220]. Recent work revealed that canonical Wnt activity drives the expansion of *isl1*-expressing cardiac progenitors and initiates the formation of pacemaker cells via Wnt5b [67,221]. The developmental origins and complexity of IFT formation and its contribution to essential cardiac functions warrant further investigation to understand its disruption in several congenital anomalies, and further studies are required to uncover the precise mechanisms driving IFT formation.

While the FHF and SHF lineages date back to the last common ancestor of chordates [19,97,184], even the ancestral purpose of forming two heart progenitor fields remains obscure. Functionally, perturbing the ratio of FHF versus SHF has revealed that their correct ratio is required to set up proper calcium conductivity for a functional myocardial syncytium [24], yet the underlying mechanisms are unknown. Overall, while previous work has provided detailed gene expression patterns of the forming heart field, integrating this knowledge into the wider CPF concept using live imaging, cell-based fate mapping, and single-cell transcriptomics will clarify the clonal lineage relationships of CPF progenitors. Live-imaging and isolation of CPF cells using transgenic zebrafish with reporters for *nkx2.5*, *gata5*, *tbx1*, and others provide a powerful basis to elucidate the lineage trajectories and differentiation of vertebrate CPF patterning. How the forelimb progenitors relate to the CPF, and if they might even be the posterior-most part of the progenitor field encompassing the CPF, also awaits clarification given the close developmental, evolutionary, and molecular connection of heart and hand [19,144,175,222].

The introduction of widely accessible genome editing to generate mutants in candidate genes of choice has further augmented the potential of zebrafish as a discovery platform for disease-causing mutations found in human patients with congenital heart anomalies. Genome sequencing has become an increasingly accessible and affordable approach for uncovering the genetic underpinnings of congenital anomalies. The rapid development and numerous readouts accumulated by the zebrafish community enable the detailed phenotype association of mutations with heart defects, as exemplified by zebrafish mutants that recapitulate key aspects of complex syndromic human disease. Of particular promise is the ability to read out phenotypes arising concomitantly with heart defects in zebrafish mutants, informing potential co-morbidities in human patients with analogous mutations.

Notably, the majority of mutations linked to CHDs have been found to be heterozygous in humans, but their orthologs behave as recessive mutants in zebrafish and also mouse models [1,223,224]. Somatic mutagenesis of candidate genes through maximized CRISPR-Cas9 efficiency enables first phenotype assessment in F0 embryos, so-called crispants [191]. Crispants enable testing of individual candidate genes or even screening [191,192,194,195,199,215]; however, the phenotypic variability observed due to incomplete saturation and mutational mosaicism warrants follow-up with germline mutants for reproductible, sustained validation (Figure 3). Further, due to the teleost-specific, additional round of whole genome duplication, mutations affecting all orthologs and possibly paralogs of a human CHD candidate gene might be needed in zebrafish at times to circumvent possibly compensatory effects. Despite these caveats, the developmental knowledge and in vivo means as outlined throughout this review render zebrafish ideally situated to act as the first discovery platform for causally connecting genetic lesions with developmental cardiac anomalies.

## Data Availability

Not applicable.

## Supplementary Materials

The following are available online https://www.mdpi.com/2308-3425/8/2/17/s1, Table S1: Zebrafish mutants of early-acting cardiac genes associated with human congenital heart disease.
