# Supplementary material for: From Stripes to a Beating Heart: Early Cardiac Development in Zebrafish
Source: J Cardiovasc Dev Dis. 2021 Feb 10;8(2):17. doi: 10.3390/jcdd8020017 (PMC7916704; doi:10.3390/jcdd8020017)
Supplement: Supplementary file 1 [file jcdd-08-00017-s001.pdf]

Table S1. Zebrafish mutants of early-acting cardiac genes associated with human congenital heart disease.

Mutant	Zebrafish Phenotype	Human Gene/Locus	Human CHD	OMIM	Refs.
van gogh(vgo); tbx1	SHF and craniofacial anomalies	*22q11*	*22q11* CNV CHD; DiGeorge Syndrome	602054	Piotrowski et al., 2003
bcl9	looping defects, valve defects, edema, arythmia	*BCL9, 1q21.2*	*1q21* CNV, Left Ventricle Hypoplasia	602597	Cantu et al., 2018
faust (fst); gata5	reduction in myocardial precursors, failed midline migration/heart tube formation	*GATA5*	multiple types of CHD	611496	Reiter et al., 1999
hands off (han); hand2	reduced myocardial progenitors, perturbed myocardial tissue	*HAND2*	Tetralogy of Fallot	602407	Yelon et al., 2000
gridlock (grl); hey2	enhanced cardiomyocyte proliferation, disrupted aortic blood flow	*HEY2*	ventricular septal defects, aortic coarctation	604674	Weinstein et al., 1995
slow muscles omitted (smu); smo	reduced cardiomyocytes, OFT formation defects, diminished endocardial morphogenesis	*SMO*	Pallister-Hall-like syndrome with CHD	601500	Barresi et al., 2000
weichers herz (whz), tbx20	decreased cardiomyocyte proliferation	*TBX20*	Atrial Septal Defect 4	606061	Just et al., 2016
heartstrings (hst); tbx5a	lack pectoral fins, slow heart rate, heart fails to loop	*TBX5*	Holt-Oram Syndrome	601620	Garrity et al., 2002
pickwick (pik), ttn	cardiomyopathy, poor contractility	*TNN, 2q31.2*	dilated cardiomyopathies	188840	Xu et al., 2002
